# Tuberculosis Vaccines: Multidimensional Exploration and Breakthroughs from Innovative Design to Evaluation Systems

**DOI:** 10.3390/vaccines13121199

**Published:** 2025-11-28

**Authors:** Wenping Gong, Ashok Aspatwar

**Affiliations:** 1 Senior Department of Tuberculosis, Chinese PLA General Hospital, Beijing 100091, China; 2 Faculty of Medicine and Health Technology, Tampere University, FI-33520 Tampere, Finland

## 1. Background

Tuberculosis is a chronic infectious disease caused by *Mycobacterium tuberculosis* (MTB) and remains a major global public health challenge [1]. According to data from the World Health Organization, there were 10.7 million new tuberculosis cases worldwide and 1.23 million deaths in 2024, making it the leading cause of death among single infectious diseases, with a mortality rate approximately twice that of HIV/AIDS [2]. Bacillus Calmette–Guérin (BCG) is currently the only approved vaccine for tuberculosis prevention. Although it has been used for more than a century, its protective effect against adult pulmonary tuberculosis is limited, and it carries potential safety risks in immunocompromised populations [3]. Therefore, developing safer and more effective new tuberculosis vaccines has become an urgent global public health priority.

Current research and development of tuberculosis vaccines primarily focus on live attenuated vaccines, subunit vaccines, viral vector vaccines, and whole-cell inactivated vaccines [3].

Although the candidate vaccine M72/AS01E has demonstrated 49.7% protective efficacy in tuberculosis prevention [4], the field still faces several key bottlenecks: a lack of standardized animal models (mouse, guinea pig, non-human primate) for evaluating vaccine efficacy, incomplete understanding of the immune protection mechanisms of vaccine candidates (including the core regulatory pathways of cellular and humoral immune responses), and substantial room for improvement in the protective efficacy of vaccines entering clinical trials. This Special Issue, entitled “Research Progress of New Tuberculosis Vaccines and Vaccine Design,” (https://www.mdpi.com/journal/vaccines/special_issues/9488WE7J90 (accessed on 14 December 2025)), includes seven high-quality research papers, covering multiple dimensions such as antigen screening and optimization, vector system innovation, evaluation model development, immune mechanism elucidation, delivery technology advancement, and adaptability studies for elderly individuals. Together, they provide a comprehensive presentation of the latest progress in tuberculosis vaccine research and offer essential theoretical support and technical foundations for continued advancements in this field.

## 2. Collection of Special Issue Articles

The first article in the Special Issue, by Huang et al., “DNA Subunit Vaccine and Recombinant BCG Based on *Mycobacterial Lipoprotein* LprO Enhance Anti-Tuberculosis Protection in the Lungs of Mice” [5] focused on the vaccine potential of the *Mycobacterium lipoprotein* LprO and successfully constructed the pcDNA-lprO DNA vaccine and recombinant BCG vaccine (BCG Japan::pNBV1-lprO). LprO, as a virulence-associated surface protein of MTB, is rich in T-cell and B-cell epitopes. Animal experiments demonstrated that both vaccines effectively induced strong Th1-type immune responses, significantly reduced MTB loads in the lungs of mice, and prolonged survival in zebrafish infected with *Mycobacterium marinum*. Importantly, the recombinant BCG Japan::pNBV1-lprO vaccine showed excellent safety in SCID mice. This study not only clarified the unique advantages of LprO as a tuberculosis vaccine antigen but also provided new technical insights for developing lipoprotein-based antigens.

The second article in the Special Issue by Ouh et al., entitled “Bacillus Calmette–Guérin Vaccination Promotes Efficient and Comprehensive Immune Modulation in Guinea Pig Models” [6], established a standardized protocol in guinea pigs (a model similar to humans in tuberculosis pathology) to reliably evaluate the immunological efficacy of BCG vaccine against MTB (H37Rv) infection. The results demonstrated that BCG vaccination four weeks prior to aerosol challenge significantly reduced the bacterial burden in the lungs and spleen compared to unvaccinated controls. Histopathological analysis revealed markedly less inflammation and lung damage in vaccinated animals. Furthermore, immunohistochemistry showed a substantial decrease in the presence of MTB bacterial loads and pro-inflammatory cytokines (TNF-α, IL-2, and IFN-γ) in the lungs of the BCG-vaccinated group. The authors conclude that their protocol provides a consistent and reliable benchmark for assessing vaccine efficacy, which can accelerate the development of safer and more effective next-generation TB vaccines.

The third article in the Special Issue, by Kozlova et al., entitled “A Cap-Optimized mRNA Encoding Multiepitope Antigen ESAT6 Induces Robust Cellular and Humoral Immune Responses Against *Mycobacterium tuberculosis*” [7], explores a cap-optimized mRNA vaccine (mEpitope-ESAT6) against MTB. The researchers tested three 5′ cap analogs in HEK293T and DC2.4 cells, finding that CapGG (a cap 1 analog) with a 4:1 cap-to-GTP ratio yielded the highest translation efficiency. In mice, the CapGG-capped vaccine induced stronger cellular and humoral immune responses than BCG, including higher IgG titers and IFN-γ production. However, despite its enhanced immunogenicity, the mRNA vaccine did not improve protective efficacy against MTB challenge, as it failed to significantly reduce bacterial loads or increase survival compared to BCG. The findings suggest that while cap optimization boosts immune responses, additional antigen components may be necessary for effective protection.

The fourth article by Pandiarajan et al. [8], entitled “Enhanced Antimicrobial Peptide Response Following Bacillus Calmette–Guerin Vaccination in Elderly Individuals”, investigated the effect of BCG vaccination on antimicrobial peptide (AMP) responses in a healthy elderly population. Plasma levels of HBD2, HNP1-3, Granulysin, and LL37 were measured at baseline, 1 month and 6 months post-vaccination. The results showed that BCG vaccination significantly increased the levels of all four AMPs in both circulating plasma and TB-antigen-stimulated plasma at 1 month, and 6 months compared to baseline. This indicates that BCG enhances both general and TB-specific innate immune responses involving these host defense peptides. No significant differences in AMP levels were found between individuals with or without LTBI. The study concludes that boosting AMP production is a potential mechanism through which BCG vaccination may enhance host defense against tuberculosis in the elderly.

The fifth article in the Special Issue, authored by de Lima et al., is entitled “Safety and Immunogenicity of an In Vivo Muscle Electroporation Delivery System for DNA-hsp65 Tuberculosis Vaccine in Cynomolgus Monkeys” [9]. This article evaluated the safety and immunogenicity of a DNA-hsp65 tuberculosis vaccine delivered via intramuscular electroporation in cynomolgus macaques. The vaccine, administered in three doses, was found to be safe, with no adverse effects on hematological, renal, or hepatic parameters, and no tuberculin skin test conversion or lung abnormalities. However, it induced only low and transient peripheral cellular immune responses and cytokine production, primarily after the third dose. The authors conclude that while electroporated DNA-hsp65 is safe, its ability to enhance cellular immunity is limited in this format. They suggest that future studies should explore combining it with cytokine plasmid adjuvants or protein prime-boost regimens to improve immunogenicity.

The sixth article in this Special Issue, authored by Jiang et al., is entitled “PP19128R, a Multiepitope Vaccine Designed to Prevent Latent Tuberculosis Infection, Induced Immune Responses in Silico and in Vitro Assays” [10]. Using bioinformatics and artificial intelligence algorithms, they screened 19 helper T lymphocyte (HTL) epitopes, 12 cytotoxic T lymphocyte (CTL) epitopes, and 8 B cell epitopes from antigens associated with latent tuberculosis infection (LTBI) and region-of-difference antigens. Then, these epitopes were fused with TLR agonists and helper peptides to construct the multiepitope vaccine PP19128R. Computational simulation predicted that this vaccine has high antigenicity and broad population coverage (82.24% for HLA class I and 93.71% for HLA class II). In vitro experiments further confirmed that PP19128R exhibits excellent antigenicity and immunogenicity, significantly increasing the number of IFN-γ–positive T lymphocytes and levels of various key cytokines, providing a novel vaccine candidate for preventing LTBI.

The seventh article in this Special Issue by Qian et al., entitled “A Multistage Antigen Complex Epera013 Promotes Efficient and Comprehensive Immune Responses in BALB/c Mice” [11], adopted a multistage composite antigen strategy, integrating three early secreted antigens (EsxH, EsxB, Ag85B) with two latency-associated antigens (nRipD, nPPE18), to construct the fusion protein vaccine Epera013f and the protein-mixed vaccine Epera013m, investigating the immunogenicity and protective efficacy of the two novel tuberculosis subunit vaccines in BALB/c mice. When formulated with an aluminum adjuvant, they induced robust humoral and cellular immune responses, as well as significant mycobacterial growth inhibition comparable to BCG. Notably, the fusion protein Epera013f elicited a more comprehensive and balanced immune response, including Th1 (IFN-γ, TNF-α), Th2 (IL-4, IL-6), and innate immunity (GM-CSF), compared to Epera013m and BCG. The findings suggest that Epera013f is a promising multistage TB vaccine candidate worthy of further development and preclinical evaluation.

## 3. Discussion

The studies featured in this Special Issue highlight key advancements and challenges in tuberculosis vaccine development. Antigen screening and structural optimization stand out as foundational, with LprO and ESAT6-based designs demonstrating strong immunogenicity, while multiepitope (PP19128R) and multistage (Epera013) antigens address broader immune targets, reducing strain escape risks. Notably, Epera013f’s balanced Th1/Th2/innate responses underscore the value of composite antigen strategies. Delivery system innovation, such as intramuscular electroporation, ensures safety but faces limitations in boosting cellular immunity, highlighting the need for adjuvant combinations or prime-boost regimens. Standardized evaluation, as established in guinea pig models, resolves inconsistencies in efficacy assessments, while studies on special populations (elderly) reveal BCG’s ability to enhance antimicrobial peptide responses, providing insights for protecting vulnerable groups. However, gaps remain: cap-optimized mRNA vaccines show enhanced immunogenicity but no improved protection, emphasizing the need for antigen composition refinement.

## 4. Conclusions

In summary, the seven studies included in this Special Issue systematically advance tuberculosis vaccine research across multiple dimensions, including antigen innovation, vaccine platform optimization, delivery system enhancement, evaluation system standardization, population-specific vaccination strategies, and in-depth immune mechanism analysis. The findings of this Special Issue provide a solid foundation for future research. Future studies should focus on multi-antigen fusion strategies, combined use of adjuvants, cross-species efficacy validation, strengthening integration between basic research and clinical translation, and promoting multicenter, large-sample preclinical and clinical trials. Additionally, interdisciplinary innovation should be emphasized, incorporating cutting-edge technologies such as artificial intelligence and gene editing throughout the vaccine development pipeline to accelerate clinical translation of new vaccines and provide strong technological support for combating the global tuberculosis epidemic.

We sincerely thank all authors, reviewers, and the editorial team for their invaluable support and hope that the research presented in this Special Issue will provide important insights for researchers and clinicians in the field of tuberculosis vaccines and help drive sustained progress.
